# *Candida auris* screening practices at healthcare facilities in the United States: A survey of the Emerging Infections Network

**DOI:** 10.1017/ash.2023.371

**Published:** 2023-09-29

**Authors:** Ian Hennessee, Kaitlin Forsberg, Susan E. Beekmann, Philip Polgreen, Jeremy Gold, Meghan Lyman

## Abstract

**Background:** *Candida auris*, an emerging fungal pathogen, is frequently drug resistant and spreads rapidly in healthcare facilities. Screening to identify patients colonized with *C. auris* can prevent further spread by prompting aggressive infection prevention and control measures. The CDC recommends *C. auris* screening based on local epidemiological conditions, patient characteristics, and facility-level risk factors; such screening might help facilities in higher burden areas to mitigate transmission and those in lower-burden areas to detect new introductions before spread begins. To describe US screening practices and challenges, we surveyed a network of infection disease practitioners, comparing responses by local *C. auris* case burdens. **Methods:** In August 2022, we emailed a survey about *C. auris* screening practices to ~3,000 members of the IDSA Emerging Infection Network. We describe survey results, stratifying findings by whether the healthcare facility was in a region where *C. auris* is frequently identified (tier 3 facility) or not frequently identified (tier 2 facility), based on CDC assessment using existing multidrug-resistant organism containment guidance (https://www.cdc.gov/hai/containment/guidelines.html). **Results:** We received 253 responses (tier 3 facilities: 119, tier 2 facilities: 134); overall, 37% performed screening. Tier 3 facilities more frequently performed screening than tier 2 facilities (59% vs 17%). Among facilities that performed screening, tier 3 facilities, compared with tier 2 facilities, more frequently screened patients on admission (84% vs 55%) and used an in-house laboratory for testing (68% vs 29%), most often with culture-based methods. Tier 2 facilities more frequently screened patients already admitted in the facility (eg, in response to cases or as part of point-prevalence surveys) compared with tier 3 facilities (59% vs 49%). Among facilities performing screening, 72% had identified ≥1 case in the previous year (tier 3 facilities, 85%; tier 2 facilities, 33%). Barriers to screening included limited laboratory capacity, long testing turnaround times, and the perception that screening was not useful. **Conclusions:** Most facilities surveyed did not perform *C. auris* screening. However, most facilities that performed screening, including those in regions of higher and lower *C. auris* burden, detected cases during the previous year. Admission screening, which might help detect new introductions before spread begins, was uncommon in facilities in lower-burden areas. Improving ease of *C. auris* screening through access to in-house laboratory testing with rapid turnaround times might increase the adoption of *C. auris* screening by facilities, thereby increasing detection and preventing spread.

**Disclosures:** None

